# How Accurate Are the Fusion of Cone-Beam CT and 3-D Stereophotographic Images?

**DOI:** 10.1371/journal.pone.0049585

**Published:** 2012-11-19

**Authors:** Yasas S. N. Jayaratne, Colman P. J. McGrath, Roger A. Zwahlen

**Affiliations:** 1 Discipline of Oral & Maxillofacial Surgery, Faculty of Dentistry, The University of Hong Kong, Hong Kong, Hong Kong SAR; 2 Discipline of Periodontology and Public Health, Faculty of Dentistry, The University of Hong Kong, Hong Kong, Hong Kong SAR; Virginia Tech, United States of America

## Abstract

**Background:**

Cone-beam Computed Tomography (CBCT) and stereophotography are two of the latest imaging modalities available for three-dimensional (3-D) visualization of craniofacial structures. However, CBCT provides only limited information on surface texture. This can be overcome by combining the bone images derived from CBCT with 3-D photographs. The objectives of this study were 1) to evaluate the feasibility of integrating 3-D Photos and CBCT images 2) to assess degree of error that may occur during the above processes and 3) to identify facial regions that would be most appropriate for 3-D image registration.

**Methodology:**

CBCT scans and stereophotographic images from 29 patients were used for this study. Two 3-D images corresponding to the skin and bone were extracted from the CBCT data. The 3-D photo was superimposed on the CBCT skin image using relatively immobile areas of the face as a reference. 3-D colour maps were used to assess the accuracy of superimposition were distance differences between the CBCT and 3-D photo were recorded as the signed average and the Root Mean Square (RMS) error.

**Principal Findings:**

The signed average and RMS of the distance differences between the registered surfaces were −0.018 (±0.129) mm and 0.739 (±0.239) mm respectively. The most errors were found in areas surrounding the lips and the eyes, while minimal errors were noted in the forehead, root of the nose and zygoma.

**Conclusions:**

CBCT and 3-D photographic data can be successfully fused with minimal errors. When compared to RMS, the signed average was found to under-represent the registration error. The virtual 3-D composite craniofacial models permit concurrent assessment of bone and soft tissues during diagnosis and treatment planning.

## Introduction

Cone-beam Computed Tomography (CBCT) and stereophotography are two of the latest imaging modalities available for three-dimensional (3-D) visualization of craniofacial structures. CBCT’s have gained popularity due to its low radiation dose, shorter scanning time and compact design. [1] Thus CBCT images are increasingly being used for diagnosis of dentofacial deformities and planning orthognathic surgery.

The main advantage of stereophotography is the visibility of natural surface colour and texture. Such information is especially beneficial when anthropometric soft tissue landmarks have to be identified or aesthetics are evaluated before or after orthognathic surgery. For example, when locating labrale superius, the boundary between the cutaneous and vermilion portion of the lip may be unclear in untextured images. [2].

**Figure 1:**
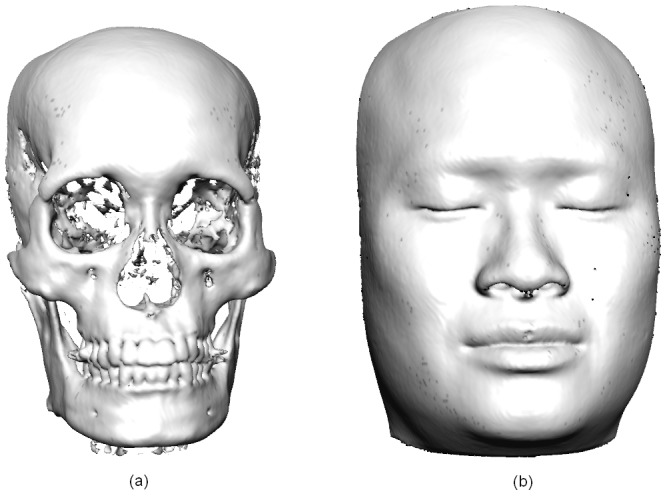
3-D hard and soft tissue images generated by Cone-beam CT.

However, each of these imaging modalities has its own advantages and limitations. Colour and texture information are not present in surface images derived from CBCT (Fig. 1). In contrast, anthropometric landmarks such as gonion or zygion cannot be detected from 3-D photographs alone as palpation is required for their accurate localization. [2] Some of these drawbacks of both techniques could be minimized by digitally combining the different image types to complement each other. Such multi-modal image fusion [3] techniques permit visualization of different tissue types as a single 3-D data set. 3-D photographs and the 3-D bone images derived from CBCT can be merged to produce a virtual craniofacial patient with natural surface texture. As the underlying bone could be visualized by altering the transparency of the surface photograph, it would be possible to identify some anthropometric landmarks which would normally require palpation. Furthermore, fusing these two image types may compensate some anatomical data which may be missed during CBCT scanning (e.g. the tip of the nose) or stereophotography (e.g. undersurface of the chin, ears).

Cephalograms and 2-D photographs were used in the initial attempts to create 3-D virtual models for craniofacial applications. [4], [5], [6], [7] Thereafter the focus moved to the possibility of merging 3-D datasets, obtained from laser scanning, CT and stereophotography. In 2001, De Groeve *et al.* [8] was able to create a photorealistic model by registering 3-D facial images acquired with a laser scanner and 3-D spiral CT images.

In 2002, Khambay *et al.* [9] conducted a validation study using a human skull embedded in Perspex covered with a latex mask to simulate the facial skin tone. Radiopaque makers were attached to the latex mask to evaluate superimposition precision between stereophotographic images and skeletal images from 3-D spiral CT scanner. They concluded that it was possible to register the 3-D photo and spiral CT data with a 1.25 - 1.5 mm accuracy.

**Figure 2:**
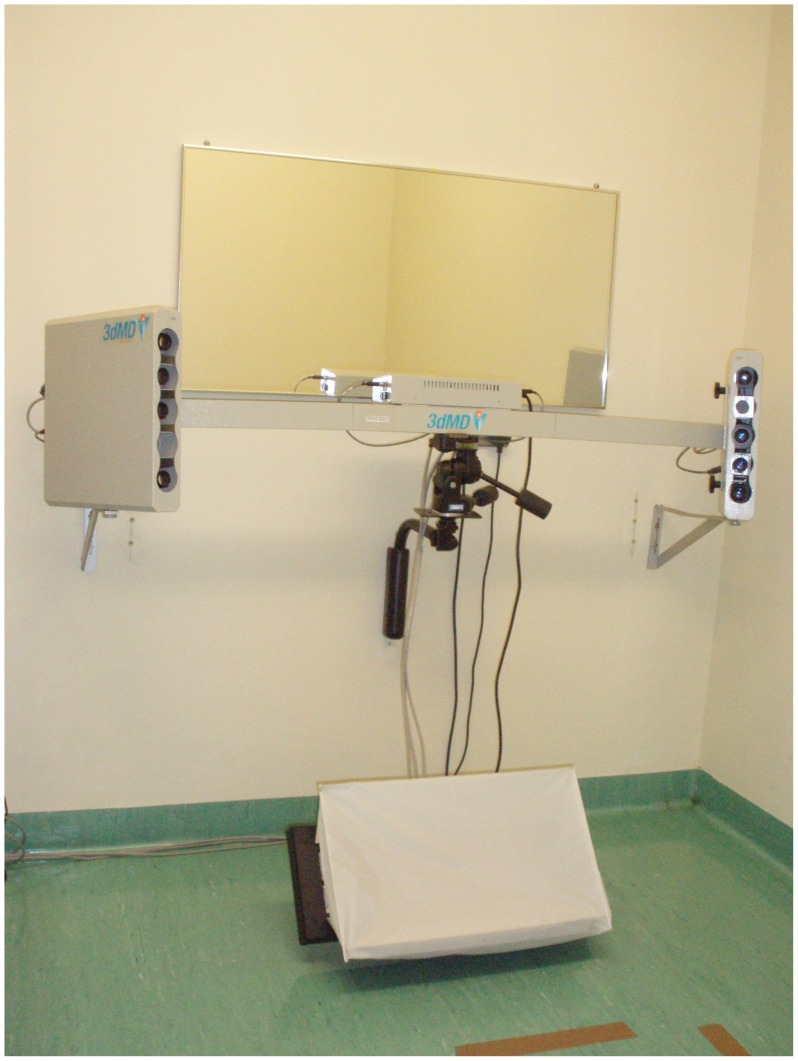
The *3dMDface* stereophotograhic system.

Ayoub *et al.* [10] replicated this study using six patients. The registration error measured as the signed average in most facial regions felt within ±1.5 mm with a maximum of 3 mm. Recently, Maal *et al.* [11] conducted a study with 15 subjects to evaluate the accuracy of superimposing 3-D photographs on skin surfaces generated from CBCT. They reported that 90% of the registration errors were within ±1.5 mm. Apart from the relatively small sample size, errors reported in all these studies were based on the average distance differences (i.e the signed average). As positive and negative values cancel each other during the calculation of the signed average, it may not be an accurate representation of the actual errors that occur during registration of two imaging modalities. Instead, the root mean square (RMS) is useful especially when distance measurements can be both positive and negative, as the errors are squared before being averaged. Therefore the objectives of this study were 1) to evaluate the feasibility of integrating 3-D Photos and CBCT images 2) to assess degree of RMS error that may occur during the above processes and 3) to identify facial regions which would be most appropriate for 3-D image registration.

**Figure 3:**
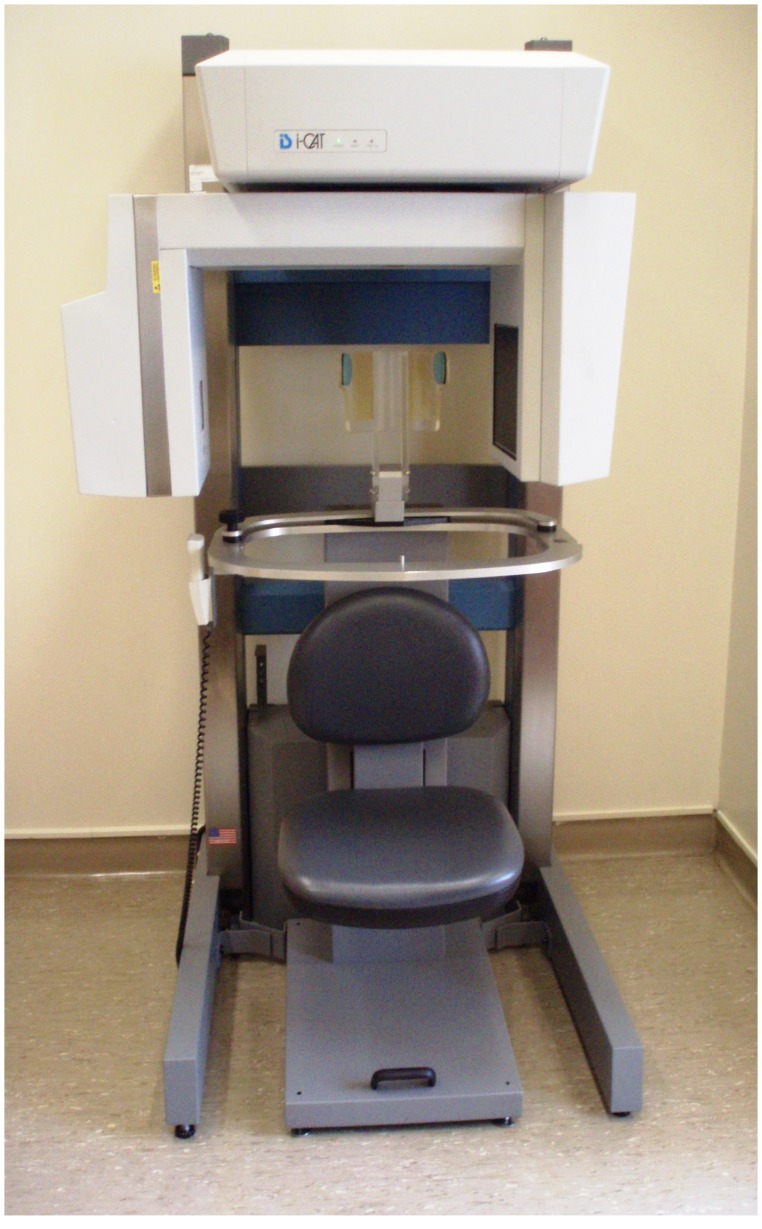
The i-CAT Cone-beam CT scanner.

## Materials and Methods

### Subjects

CBCT scans and stereophotographic images from 29 patients were used for this study. These images were acquired as a part of their normal orthognathic surgical treatment protocol.

**Figure 4:**
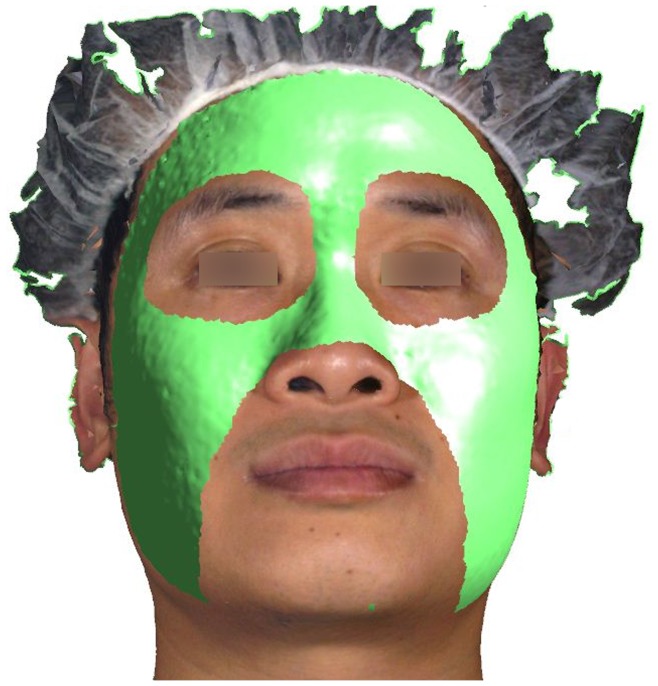
The region used as a reference during registration.

**Figure 5:**
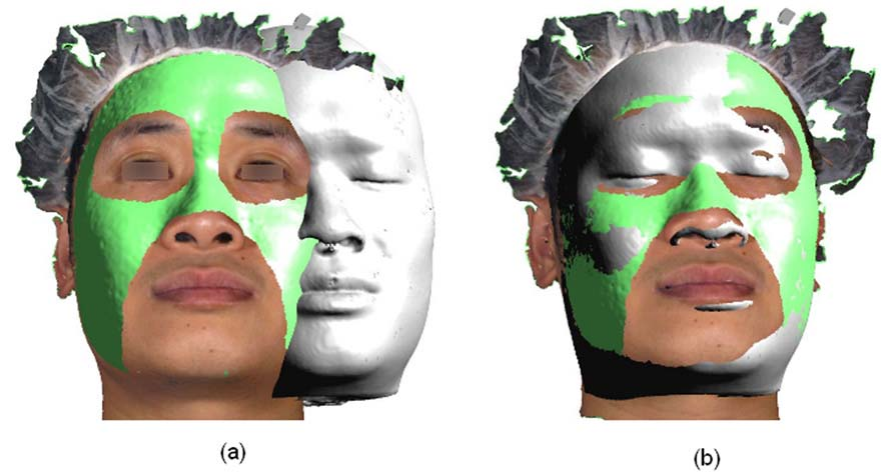
The matching of Cone-beam CT soft tissue and stereophotograhic images during registration. Initial manual aligning (a) is followed by automated registration (b).

### Ethics Statement

This research project was approved by the Institutional Review Board (IRB) of The University of Hong Kong/Hospital Authority Hong Kong West Cluster (Protocol No: UW 12-066). As these images were obtained as a part of the routine treatment protocol and due to the retrospective nature of this study, a waiver of consent was granted by the IRB. The subject depicted in the figures has given informed consent for publication of his photograph.

**Figure 6:**
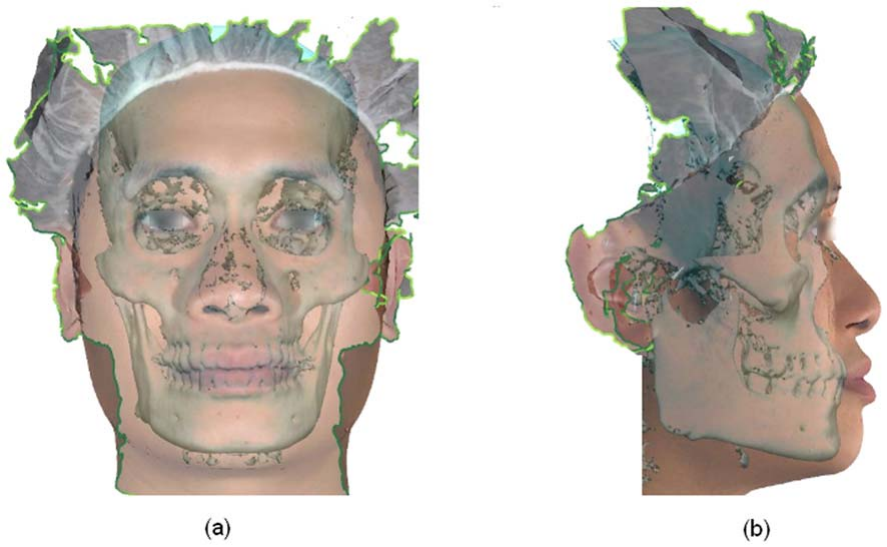
The frontal (a) and lateral (b) views of the virtual craniofacial model generated by fusing the Cone-beam CT and stereophotographic images.

### Image Acquisition

The 3-D photographs were acquired with the *3dMDface* stereophotography system (3dMD, Atlanta, USA). This system consisted consist of two pods separated by a known base distance (Fig. 2). Each pod contains three machine vision cameras (two monochrome and a colour camera) with specific angulations. A random pattern of light is projected on the subject during image acquisition and overlapping images (four grayscale and two colour) are simultaneously captured. A stereo-triangulation algorithm is used to generate the 3-D geometry by matching the correspondence between acquired gray scale image pairs. The colour and texture information is then mapped on to the 3-D model. [12] The *3dMDface* system has been validated in terms of its accuracy and reliability. [13],[14] Patients were seated in a chair placed at a set distance from the cameras while looking at a mirror placed in front of them. They were instructed to maintain a natural pose with neutral expression.

**Figure 7:**
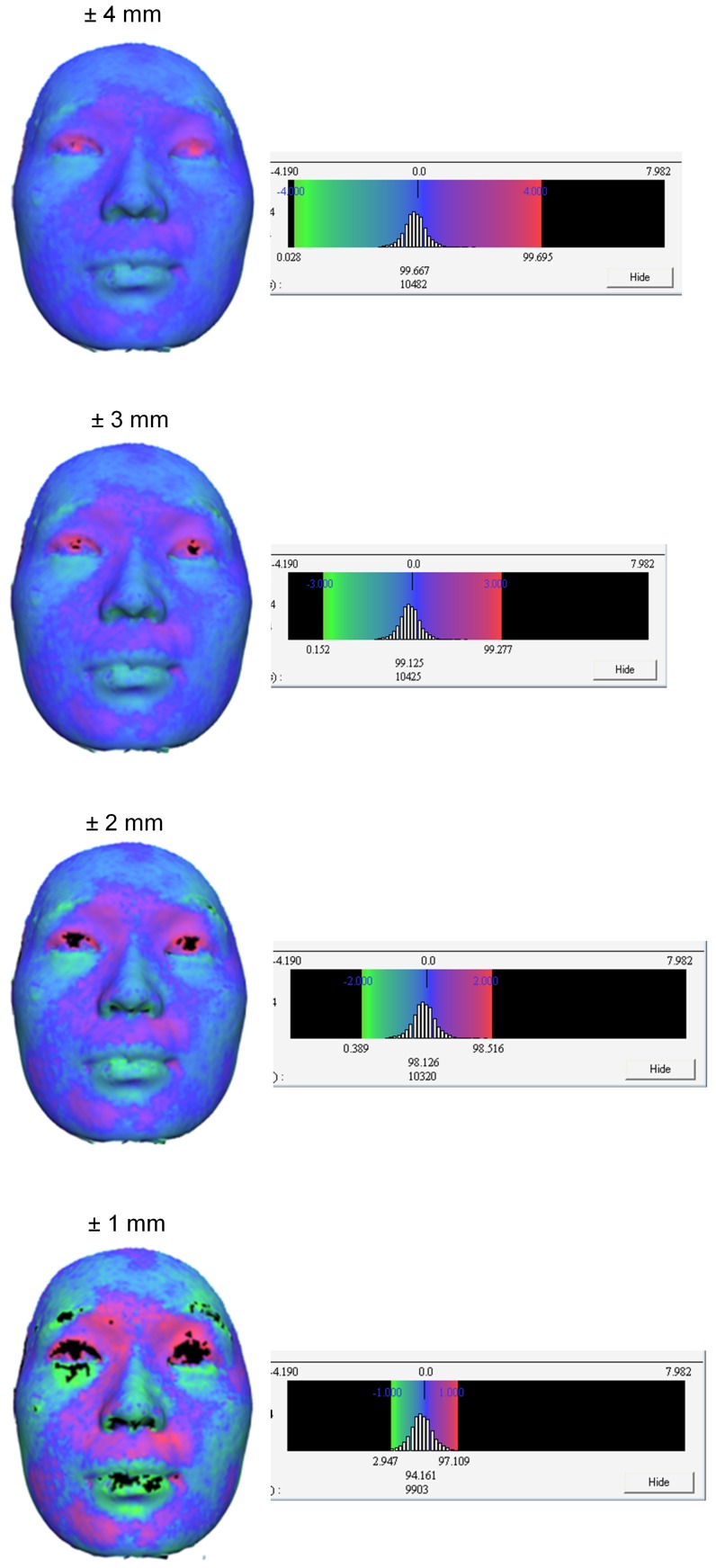
Colour maps and the accompanying histogram. The ± sign depicts the distance range of the histogram.

Following 3-D photography each subject underwent a CBCT scan (i-CAT System, Imaging Sciences International, Hatfield, PA, USA) with the full field of view (16 cm×13 cm). The CBCT system (Fig. 3) employs a cone shaped beam of X-rays which captures the full volume of interest in a single rotation. The technique in CBCT differs from traditional medical CT scanners which uses a fan shaped beam that images the subject as sequential slices. The flat panel detector and the X-ray source rotate simultaneously while the patient remains stationary.

**Table 1:** Registration errors and distance differences between superimposed CBCT and 3-D photographic skin surfaces.

Subject No:	RMS error forregistration (mm)	Signed average of differencebetween surfaces (mm)	RMS value of differencebetween surfaces (mm)
**1**	0.581	0.069	1.272
**2**	0.498	0.108	0.905
**3**	0.316	0.042	0.579
**4**	0.545	0.224	1.192
**5**	0.480	0.149	0.983
**6**	0.310	−0.041	0.674
**7**	0.462	−0.064	0.661
**8**	0.494	−0.018	0.644
**9**	0.392	−0.080	0.678
**10**	0.497	0.017	0.702
**11**	0.312	−0.080	0.581
**12**	0.382	−0.075	0.630
**13**	0.388	0.009	0.745
**14**	0.407	−0.039	0.615
**15**	0.291	−0.015	0.540
**16**	0.494	−0.290	1.300
**17**	0.490	0.155	0.711
**18**	0.751	−0.178	1.035
**19**	0.365	0.099	0.687
**20**	0.539	−0.215	0.881
**21**	0.430	0.147	0.711
**22**	0.240	−0.036	0.515
**23**	0.421	−0.033	0.434
**24**	0.844	−0.240	1.033
**25**	0.308	−0.123	0.448
**26**	0.406	0.154	0.669
**27**	0.469	0.068	0.586
**28**	0.316	−0.038	0.453
**29**	0.371	−0.196	0.579
**Mean**	0.441	−0.018	0.739
**SD**	0.131	0.129	0.239
**Median**	0.421	−0.033	0.674
**1^st^ Quartile**	0.365	–0.080	0.581
**3^rd^ Quartile**	0.494	0.069	0.881

During CBCT scanning patients were instructed to remain still while breathing normally through the nose. The chin cap was not used as it can distort soft tissues. Each scan was stored in the Digital Imaging and Communications in Medicine (DICOM) multi-file format for further processing.

**Figure 8:**
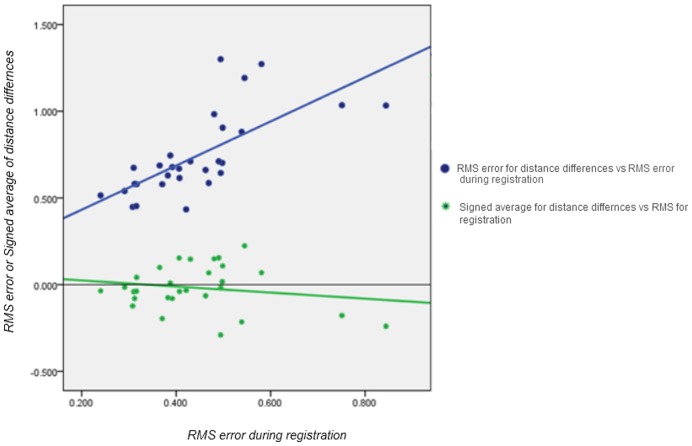
Scatter plot of the relationship between measurements used for error analysis.

### Image Processing

A software program called “Multi File Converter” provided by the manufacturer was used to convert the DICOM multi-file format files into the binary format, in order to be read by the *3dMDpatien*t software (3dMD LLC, Atlanta, USA).

Once the binary file was imported, the software extracted two 3-D images corresponding to the skin and bone from the CBCT data. This was possible as the software is able to analyze the Hounsfield Units and recognize the bone and skin surface from the CBCT scan. These two images were saved as.tsb files. Then the CBCT skin and bone images were loaded to the software. These images align automatically as both are from the same capture session and modality. The 3-D photograph was loaded thereafter and manually approximated over the CBCT skin image.

Areas of the face other than periorbital and nasolabial regions were selected on each 3-D photo to be used as a reference during registration (Fig. 4). These two regions were excluded because they are much more affected during facial expressions. [15] Using the selected area as reference, the 3-D photo was registered on the CBCT skin image. Initially the two surfaces were aligned manually. Thereafter the registration software was evoked and the alignment process continued automatically to achieve optimum registration by minimizing the distance between the two superimposed surfaces (Fig. 5). The software employs a modified version of the Iterative Closest Point (ICP) algorithm [16] for performing surface-based registration.

The Root Mean Square (RMS) error during each registration was recorded. Thereafter the 3-D photograph and the 3-D skull model were saved as composite image, resulting in a virtual craniofacial model (Fig. 6).

### Generation of Colour Maps

3-D colour maps were used to assess the accuracy of registration of 3-D photograph and the CBCT skin images. The technical details of creating these maps have been explained in detail in a paper already published elsewhere. [17] The whole surface of the face was selected as the region of interest. The following information which indicates the discrepancy between the two registered surfaces was recorded from the histogram accompanying the colour map for each virtual model.

1. The signed average of the distance measurements
2. The Root Mean Square (RMS) value of the distance measurements

The signed average is computed by the software by simply adding up positive and/or negative distance differences and calculating their absolute mean. RMS value can be used to account for the effect of positive and negative signs. The computation of RMS involves the following steps: 1) all values are squared; 2) the squares are added together and its average is calculated; 3) the square root of the resulting average is estimated. The RMS value can be represented by the following formula:
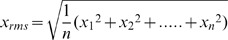



In order to identify the regions of the face contributing to most errors, the upper and the lower limit of the colour maps were reduced at 1 mm increments from +/−4 mm (Fig. 7). This process enhances the visualization of most incompatible regions.

### Statistical Analysis

Descriptive statistics including the mean, SD, median, and interquartile range were calculated for each subject. Based on the results of the Shapiro-Wilk test for normality, the Friedman two way ANOVA test was performed to compare the three interrelated methods used for error analysis with the null hypothesis being that the distribution of RMS Error for Registration, Signed average and RMS value of difference between surfaces were the same. The Spearman rank correlation was calculated to explore the relationship between the RMS value during registration and the distance difference between the two surfaces indicated in the colour maps. These statistical tests were performed with PASW 18 software (SPSS, Chicago, IL, USA).

## Results

The results obtained for each subject are presented in Table 1. The mean RMS for registration was 0.441 (±0.131) mm, while the mean values for the signed average and RMS of the distance differences between the registered surfaces were −0.018 (±0.129) mm and 0.739 (±0.239) mm respectively. The result of the Friedman test was p<0.001, indicating significant differences among the three methods.

The Spearman’s correlation (Fig. 8) between the RMS value during registration and the RMS of the distance differences depicted in the colour map was 0.771 mm (p<0.001). The same correlation coefficient for the relationship between RMS during registration and the signed average for the distance differences depicted in the colour map was 0.094 mm (p = 0.626). The most variable regions in the face during registration recognized by narrowing the scale of the colour maps were areas surrounding the lips and the eyes. In contrast, the regions overlying the forehead, root of the nose and zygoma proved to be quite stable.

## Discussion

This study demonstrated the feasibility of creating photorealistic craniofacial virtual models with minimum errors. The mean RMS error of 0.739 mm for the whole face after superimposing the 3-D photograph on the CBCT skin image was much better than the acceptable limit of 2 mm recommended by the software developer.

The mean RMS error during registration (0.441 mm) was significantly lower when compared to RMS values obtained from colour maps. It may be assumed that this occurs because the reference regions used for registration were much more stable, resulting in a more accurate registration and lower RMS values. In contrast, the RMS error increases when the whole facial surface is included in the colour map. This can be due to more matching errors resulting between the two surfaces, due to unstable perioral and orbital regions leading to a higher RMS value. The correlation between RMS error during registration and the whole facial surface measured from colour maps was high, illustrating the importance of achieving a low RMS error during registration.

Evaluation of the colour maps revealed that regions of the face with minimal skin laxity such as forehead, nasal root and zygoma were relatively static. In addition, these areas usually remain unaltered following orthognathic surgery. Thus, these regions would be preferable for use as reference regions when superimposing 3-D images to perform longitudinal assessment of treatment outcomes. Ayoub *et al*. [10] has previously reported that relatively large errors were found around the cheeks, eyelids and eyebrows that when registering 3-D photo and spiral CT images. The error around cheeks may have occurred due soft tissue drape resulting from gravity while spiral CT scans are performed in the supine position. As CBCT is carried out in an upright posture, such marked errors in the cheek region were not encountered in the current study.

Ayoub *et al.* [10] reported errors within ±1.5 mm with a maximum of 3 mm for registering spiral CT images with stereophotographs while Maal *et al.* [11] found that 90% of the registration errors were within ±1.5 mm. However, both studies have reported their results using the signed average. As the positive and negative values cancel each other when computing the signed average, this metric gives quite low error values compared to the RMS. For example, in the current study the mean RMS error for the whole face was 0.739 mm whereas the signed average was only −0.018 mm. Thus the actual errors between the two superimposed surfaces may have been underrepresented in previous studies [9],[10],[11] using the signed average to report their results..

Both bone and soft tissues are visible as a single 3-D image in the created virtual models. Therefore it is possible to simultaneously assess both tissue types individually as well as their interrelationships. These virtual models can also be used for the estimation of soft tissue thicknesses corresponding to the underlying bone in facial regions. Such information would of value for forensic reconstructions based on skeletal remains.

Fusion of two imaging modalities also helps to overcome surface artifacts caused by patient movement as well as missing anatomical data. Sometimes the tip of the nose or forehead may not be captured in CBCT scans due to limitations in the field of view. In addition, stabilization devices such as chin rests and head bands may be required especially for children undergoing CBCT scanning. These devices compress soft tissues and alter the normal morphology. On the other hand, areas such as the ears and submental region are often missed in 3-D photographs. Thus, combining the two imaging modalities compensates for such drawbacks.

Virtual craniofacial models also facilitate evaluation of treatment outcomes. The pre and postoperative models can be superimposed using the cranial base as a reference. Afterwards, the hard and soft tissue changes resulting from surgery can be quantified. Accurate fusion enables correlation soft tissue changes with the degree of bone movement during orthognathic surgery. Therefore, generation of accurate virtual craniofacial models would be the first step in creating 3-D real-time surgical simulation algorithms and software.

The inability to replicate the same facial expression during the two image acquisition sessions may be the major factor contributing to errors during superimposition of CBCT and 3-D photographic data. The CBCT scanning takes 40 seconds versus 2 milliseconds for capturing a 3-D photograph. Therefore the chance for facial muscle movement due to breathing, swallowing as well as changes of head posture during CBCT is quite high. All these changes will contribute to discrepancies during superimposition. [18] Integrating a 3-D photographic camera into the CBCT scanner might be a plausible option to overcome this problem. Such simultaneous recording of hard and soft tissue data would greatly help in minimizing errors which occur during the creation of virtual craniofacial models.

### Conclusion

The results of this study demonstrate the feasibility of integrating CBCT and 3-D photographic data with minimal errors. When compared to RMS, the signed average was found to under-represent the superimposition error. The virtual 3-D composite craniofacial models permit concurrent assessment of bone and soft tissues. These models can be used as an objective tool for diagnosis and treatment planning in orthognathic surgery.

## References

[1] Jayaratne YS, Zwahlen RA, Lo J, Tam SC, Cheung LK (2010) Computer-aided maxillofacial surgery: an update. Surg Innov 17: 217–225. 20513723 10.1177/1553350610371626

[2] Jayaratne YS, Zwahlen RA, Cheung LK (2010) Re: Three-dimensional anthropometric analysis of the Chinese nose. J Plast Reconstr Aesthet Surg 63: 1840–1841. 20395186 10.1016/j.bjps.2010.03.013

[3] Vannier M, Gayou D (1988) Automated registration of multimodality images. Radiology 169: 860. 3263667 10.1148/radiology.169.3.3263667

[4] Curry S, Baumrind S, Carlson S, Beers A, Boyd R (2001) Integrated three-dimensional craniofacial mapping at the Craniofacial Research Instrumentation Laboratory/University of the Pacific. Seminars in Orthodontics 7: 258–265.

[5] Grayson B, Cutting C, Bookstein FL, Kim H, McCarthy JG (1988) The three-dimensional cephalogram: Theory, techniques, and clinical application. American Journal of Orthodontics and Dentofacial Orthopedics 94: 327–337. 3177285 10.1016/0889-5406(88)90058-3

[6] Ip HHS, Yin L (1996) Constructing a 3D individualized head model from two orthogonal views. Visual Computer 12: 254–266.

[7] Xia J, Wang D, Samman N, Yeung RWK, Tideman H (2000) Computer-assisted three-dimensional surgical planning and simulation: 3D color facial model generation. International Journal of Oral and Maxillofacial Surgery 29: 2–10. 10691135

[8] De Groeve P, Schutyser F, Van Cleynenbreugel J, Suetens P (2001) Registration of 3D photographs with spiral CT images for soft tissue simulation in maxillofacial surgery. Lecture Notes in Computer Science 2208: 991–996.

[9] Khambay B, Nebel JC, Bowman J, Walker F, Hadley DM, et al (2002) 3D stereophotogrammetric image superimposition onto 3D CT scan images: the future of orthognathic surgery. A pilot study. The International journal of adult orthodontics and orthognathic surgery 17: 331–341. 12593005

[10] Ayoub AF, Xiao Y, Khambay B, Siebert JP, Hadley D (2007) Towards building a photo-realistic virtual human face for craniomaxillofacial diagnosis and treatment planning. International Journal of Oral and Maxillofacial Surgery 36: 423–428. 17428638 10.1016/j.ijom.2007.02.003

[11] Maal TJ, Plooij JM, Rangel FA, Mollemans W, Schutyser FA, et al (2008) The accuracy of matching three-dimensional photographs with skin surfaces derived from cone-beam computed tomography. International Journal of Oral and Maxillofacial Surgery 37: 641–646. 18539435 10.1016/j.ijom.2008.04.012

[12] Lane C, Harrell W Jr (2008) Completing the 3-dimensional picture. American journal of orthodontics and dentofacial orthopedics : official publication of the American Association of Orthodontists, its constituent societies, and the American Board of Orthodontics 133: 612–620. 10.1016/j.ajodo.2007.03.023 18405826

[13] Weinberg SM, Naidoo S, Govier DP, Martin RA, Kane AA, et al (2006) Anthropometric precision and accuracy of digital three-dimensional photogrammetry: comparing the Genex and 3dMD imaging systems with one another and with direct anthropometry. J Craniofac Surg 17: 477–483. 16770184 10.1097/00001665-200605000-00015

[14] Aldridge K, Boyadjiev SA, Capone GT, DeLeon VB, Richtsmeier JT (2005) Precision and Error of Three-Dimensional Phenotypic Measures Acquired From 3dMD Photogrammetric Images. American journal of medical genetics 138: 247–253. 10.1002/ajmg.a.30959 PMC4443686 16158436

[15] Trotman CA, Faraway JJ, Silvester KT, Greenlee GM, Johnston LE Jr (1998) Sensitivity of a method for the analysis of facial mobility. I. Vector of displacement. Cleft Palate Craniofac J 35: 132–141. 9527310 10.1597/1545-1569_1998_035_0132_soamft_2.3.co_2

[16] Besl PJ, McKay ND (1992) A method for registration of 3-D shapes. IEEE Transactions on Pattern Analysis and Machine Intelligence 14: 239–256.

[17] Jayaratne YS, Zwahlen RA, Lo J, Cheung LK (2010) Three-dimensional color maps: a novel tool for assessing craniofacial changes. Surg Innov 17: 198–205. 20542953 10.1177/1553350610370752

[18] Schendel SA, Duncan KS, Lane C (2011) Image fusion in preoperative planning. Facial plastic surgery clinics of North America 19: 577–590. 22004853 10.1016/j.fsc.2011.07.002

